# Field Applications of Fluorinated Nematicides for *Meloidogyne enterolobii* Management on Tomato

**DOI:** 10.2478/jofnem-2024-0030

**Published:** 2024-08-16

**Authors:** R. Castro-López, C. A. López-Orona, J. A. Martínez-Gallardo, M. A. Tirado-Ramírez, G. Gómez, W. Rubio-Aragón, J. A. Edeza-Urias, M. C. Villa-Medina

**Affiliations:** Facultad de Agronomía, Universidad Autónoma de Sinaloa, Culiacán, Sinaloa, 80000, México

**Keywords:** *M. enterolobii*, fluopyram, fluensulfone, fluazaindolizine, root-knot nematode, field evaluation, nematicide interactions, management, chemical control

## Abstract

Mexico is the 8^th^ largest producer of tomatoes. *Meloidogyne enterolobii* is reported in Sinaloa, affecting tomato cultivars with genetic resistance to *Meloidogyne* spp. We aimed to evaluate field applications of fluopyram, fluensulfone, and fluazaindolizine treatments for managing *M. enterolobii* on tomatoes. Experiments were set on raised beds in a shade house. Nematicides were applied via drip irrigation. Under fluopyram treatment, *M. enterolobii* did not reduce the number of extra-large-size fruits. The number of large-size fruits with fluopyram and fluazaindolizine plus fluopyram treatments was also unaffected by *M. enterolobii*. Yield from the treatments fluopyram, fluazaindolizine plus fluopyram, and fluensulfone plus fluopyram was similar to the control treatment without *M. enterolobii*. Finally, fluazaindolizine plus fluopyram, fluopyram, and fluensulfone plus fluopyram treatments showed the highest reduction of root galling. We conclude that the fluopyram was more effective as an individual treatment. Pre-plant applications of fluensulfone and fluazaindolizine reduced the damage to the plant and the loss of yield; however, the complementary application of fluorinated nematicides improved the management of *M. enterolobii* in the tomato crop.

Tomato is a primary food source worldwide. Mexico is the 8^th^ largest producer of tomatoes, producing more than four million tons per year (Faostat, 2021). Twenty-seven percent of Mexican tomatoes are grown in Sinaloa; however, the root-knot nematode (RKN) *Meloidogyne* spp. is a worrying factor that threatens vegetable production, including tomatoes in Sinaloa. The pacara earpod tree root-knot nematode, *Meloidogyne enterolobii*, (Yang and Eisenback, 1983) is characterized by its elevated pathogenicity and ability to overcome resistance. *Meloidogyne enterolobii* was previously reported in Sinaloa, affecting a tomato cultivar with genetic resistance to *M. incognita* and *M. javanica* (Martínez et al., 2015). Subsequently, this species was reported to induce severe root galling from a low inoculum level on a short-cycle crop such as cucumber (Gómez-González et al., 2020). Currently, resistant cultivars are not commercially available; therefore, control programs are limited to keep the population levels of *M. enterolobii* in soil under the economic threshold of damage (Rashidifard, 2019). Thus, developing different strategies to increase effectiveness in nematode management programs is crucial.

In Sinaloa, RKN management mainly utilizes the nematicide groups organophosphate and carbamates; these classic nematicides are characterized by their high toxicity to non-target organisms, including humans (Oka, 2020). Therefore, the restriction on using these nematicides has increased in recent years. Safer and selective (next-generation) nematicides such as fluazaindolizine [8-chloro-N-(2-chloro-5-methoxyphenyl)sulfonyl-6-(trifluoromethyl) imidazo[1,2-a]pyridine-2-carboxamide], fluensulfone [5-chloro-2-(3,4,4-trifluorobut-3-enylsulfonyl)-1,3-thiazole], and fluopyram [N-[2-[3-chloro-5-(trifluoromethyl)-2-pyridinyl]ethyl]-2-(trifluoromethyl)benzamide], have been recently developed and tested with promising results. These novel compounds contain the functional trifluoromethyl (-CF_3_) group (fluorinated nematicides). Incorporating -CF_3_ in compounds enhances chemical and metabolic stability, improves lipophilicity and bioavailability, and increases the protein binding affinity (Chu and Qing, 2014). The modes of action of fluazaindolizine and fluensulfone are unknown. Fluazaindolizine severely affects muscle motion and causes a cessation of feeding, paralysis, and nematode death (Chen et al., 2018). The effects of fluensulfone on motility and body posture are similar to organophosphates, carbamates, and avermectins; however, fluensulfone has a broad range of effects on the reproduction, development, and feeding of nematodes (Kearn et al., 2014). Fluopyram is a succinate dehydrogenase inhibitor (SDHI) (Avenot and Michailides, 2010). Specifically, fluopyram inhibits the mitochondrial complex II of the aerobic respiratory chain (Schleker et al., 2022). Fluopyram has been found to induce reversible paralysis in plant-parasitic nematodes (Faske and Hurd, 2015).

Adverse physicochemical conditions in the soil, microbes that degrade nematicides, and leaching of nematicides reduce nematicide efficacy (Oka, 2020). Moreover, sub-lethal dosages lessen the efficacy of nematicides and generate selection pressure, potentially triggering resistance development (Meher et al., 2009). Therefore, field evaluations are imperative since they contribute information on the effects of the compounds in interaction with the agro-environmental conditions. Likewise, field evaluations allow the adaptation of the application timings regarding the duration of the nematicidal activity in the soil, avoiding sub-lethal dosages. Several studies have evaluated next-generation nematicides on major plant-parasitic nematodes, contributing to valuable data for nematode management (Desaeger et al., 2019). However, data on the effects of combinations and application timings of fluorinated nematicides against *M. enterolobii* is still scarce. Therefore, this study aimed to evaluate the impact of field applications of fluazaindolizine, fluensulfone, and fluopyram for managing *M. enterolobii* on tomato crops.

## Materials and Methods

### Isolation and identification of *M. enterolobii*

**Figure S1.**
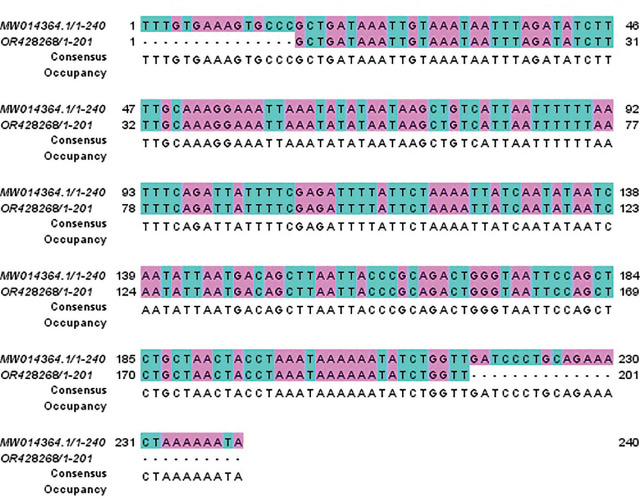
Alignment of sequences OR428268 and MW014364.1 of *M. enterolobii* intergenomic spacer 5S–18S rRNA. Alignment was performed with the MAFFT sequence alignment server (https://mafft.cbrc.jp/alignment/server).

In February, 2020, galled eggplant roots were obtained from a local field (24.255509, -107.185518). Nematode females (n=30) and their egg masses were dissected and individually recovered in labeled microcentrifuge tubes. Each egg mass was individually used to inoculate tomato (cv. Cuauhtémoc) seedlings (4-week-old). Molecular identification was conducted on each female with the NaOH method (Hu et al., 2011). The species-specific primers Me-F/Me-R (Long et al., 2006) and Mi-F/Mi-R (Meng et al., 2004) were employed. Fragments of ≈230 bp were amplified in all reactions (n=30) according to the correct identification of *M. enterolobii*. No fragments were amplified with Mi-F/Mi-R primers. The seedlings (n=30) infected with egg masses from *M. enterolobii*-positive females were individually established in 15 L pots. Pots were filled with sterilized clay loam soil. The tomato seedlings were allowed to grow in a shade house for six months. Then, molecular re-identification was performed on a female from each pot (n=30). All reactions amplified fragments of ≈230 bp with the species-specific primers Me-F/Me-R. On this occasion, a representative amplicon was purified with the Wizard Genomic DNA Purification Kit (Promega, Madison, WI) and sequenced at IPICYT (SLP, Mexico). The BLAST server conducted the sequence homology search at the NCBI. It was found to have 100% identity and query coverage with *M. enterolobii* (MW014364.1). Aligned sequences are shown in Supplementary Fig. 1. The sequence from our isolate was deposited in the NCBI GenBank database (OR428268). Once *M. enterolobii* was confirmed, roots were extracted from plots and washed thoroughly. Root samples (20 g) were taken to determine the egg population per gram of root (Gómez-González et al., 2021). Soil samples (200 cm^3^) were also processed by sieve-funnel technique to determine the juvenile J2 population levels (Hooper et al., 2005). The subsequent experiments to evaluate nematicides were infested with these roots and soil.

**Figure 1.**
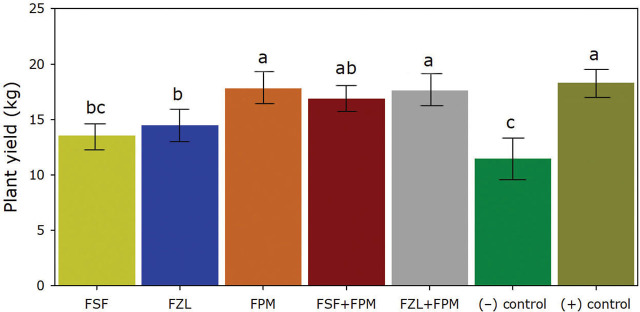
Size of fruits of tomato plants under fluorinated nematicide treatments for managing *Meloidogyne enterolobii*. FSF= fluensulfone, FZL= fluazaindolizine, FPM= fluopyram, FSF+FPM= fluensulfone plus fluopyram, FZL+FPM= fluazaindolizine plus fluopyram, (−) control= inoculated plants without nematicide treatment, (+) control= non-inoculated plants without nematicide treatment. Data are means ± standard errors of 168 plants per treatment. Bars (within the same fruit size group) with different lower-case letters indicate significant differences (*P* ≤ 0.05) according to the Tukey test.

### Field experiments

The experiments were carried out in a shade house in the 2020–2021 crop season and repeated in 2021–2022 under similar conditions. Fourteen soil beds (7-m-long) were raised with a row center spacing of 1.8 m. Soil composition was 45% silt, 35% clay, and 20% sand (pH 6.8). Beds were arranged in a randomized complete block design, and two beds were randomly assigned for each treatment. Nematode infestation was carried out on twelve beds. The inoculum was 20 g of tomato-galled roots (≈96,000 eggs) and, 200 cm^3^ of infested soil (≈2,400 juveniles J2) per each 20 linear cm. The soil was thoroughly mixed, and the drip tape and plastic mulch were placed. Beds were immediately watered (5 L/linear meter).

At 17 days after inoculation, tomato (cv. Cuauhtemoc) seedlings (45 days old) were transplanted into the beds in single rows, with 25 cm within row spacing (28 plants/bed). Tensiometers 2725 ARL (Soil Moisture Crop; USA) were installed at a depth of 15 cm. Beds were irrigated in the morning if tensiometers reached −20 kPa of soil water potential. Nutrient solutions were composed according to Steiner (1966).

Beds were watered (1 L/linear meter) as pretreatment irrigation. Then, nematicides were dissolved in water and injected into the drip system (5 L/linear meter), followed by a final watering (1 L/linear meter). Employed nematicides were: fluensulfone (Nimitz, a.i. 48%, ADAMA, Raleigh, NC), fluazaindolizine (Salibro, a.i. 41%, Corteva, Indianapolis, IN), and fluopyram (Verango, a.i. 50%, Bayer, Research Triangle Park, NC). Nematicides were applied according to manufacturer suggestions at maximum rates of active ingredients (a.i.) for a nematicidal activity period in the soil of no more than 50% of the crop cycle (210 days). Nematicide treatments were: FSF (960 g of a.i./ha of fluensulfone at 12 days before planting [DBP]), FZL (820 g of a.i./ha of fluazaindolizine at 7 DBP), FPM (500 g of a.i./ha of fluopyram at 15 and 40 days after planting [DAP], FSF+FPM (960 g of a.i./ha of fluensulfone at 12 days DBP plus 500 g of a.i./ha of fluopyram at 35 and 60 DAP), and FZL+FPM (820 g of a.i./ha of fluazaindolizine at 7 DBP plus 500 g of a.i./ha of fluopyram at 35 and 60 DAP). In treatments that involved pre-plant nematicide and additional fluopyram applications, the application timing of the latter was precautionarily modified to avoid phytotoxicity. Final nematicide treatments, application timings, and dosages are described in Table 1.

**Table 1.** Treatments of fluorinated nematicides for the management of *Meloidogyne enterolobii* on tomato.

**Treatment**	**Active ingredient**	**Application timings^a^**	**a.i./ha^b^**
FSF	Fluensulfone	12 DBP	960 g
FZL	Fluazaindolizine	7 DBP	820 g
FPM	Fluopyram	15 and 40 DAP	1.0 kg
FSF+FPM	Fluensulfone	12 DBP	960 g
	Fluopyram	35 and 60 DAP	1.0 kg
FZL+FPM	Fluazaindolizine	7 DBP	820 g
	Fluopyram	35 and 60 DAP	1.0 kg

a DBP=days before planting, DAP=days after planting.

b Total amount applied of each active ingredient.

### Variables and statistical analyses

The number of plants was 56 per treatment in each experiment. Plants from beds without nematode inoculation were used as the positive control. Plants from inoculated beds without nematicide treatment were used as the negative control. The fruit harvest was carried out at 3–5 days intervals, starting at 71 DAP and ending at, 200 DAP. All fruits were counted, weighted, and classified by size according to USDA (1991) grades: small 5.4 to 5.6 cm, medium 5.7 to 6.3, large 6.4 to 7.0 cm, and extra-large 7.1 or higher sizes. The plant yield was the sum of weight from all fruits per treatment. At the end of the experiment (210 DAP), all plants were cut at the soil level and evaluated for plant height, stem diameter, and plant fresh weight. Roots were extracted from the soil and washed thoroughly. Percentages of galling were then estimated on each root using a 10% scale (Garabedian and Van Gundy, 1983). Finally, roots were dried in an air oven at 60°C for 48 h and weighed. Data from each variable were subjected to ANOVA to determine the differences among experiments. Data were analyzed using the Proc GLM for a one-way ANOVA and with the Tukey test (α = 0.05) to detect significant differences among treatments (SAS v.9.1) (SAS Institute, Cary, NC).

## Results

Data from both experiments were similar and were combined. The yield from tomato plants under nematicide treatments FZL+FPM, FPM, and FSF+FPM was similar (*P* > 0.05) when compared with the non-inoculated control (18.33 kg). The observed yield with FZL and FSF was lower (*P* ≤ 0.05) when compared with FPM and FZL+FPM treatments (17.83 and 17.62 kg, respectively). The yield from plants under FSF treatment was similar (*P* > 0.05) to the inoculated control without nematicide application (11.49 kg) (Fig. 1).

**Figure 2.**
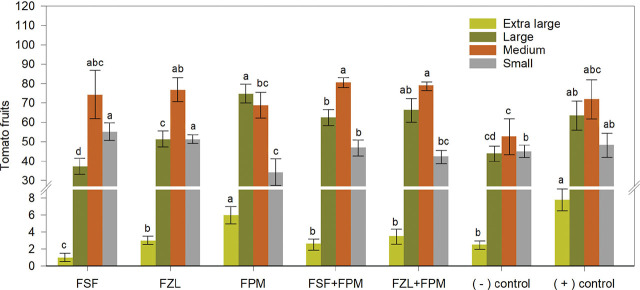
Plant yield of tomato plants under fluorinated nematicide treatments for managing *Meloidogyne enterolobii*. FSF= fluensulfone, FZL= fluazaindolizine, FPM= fluopyram, FSF+FPM= fluensulfone plus fluopyram, FZL+FPM= fluazaindolizine plus fluopyram, (+) control= non-inoculated plants without nematicide treatment, (−) control= inoculated plants without nematicide treatment. Data are means ± standard errors of 168 plants per treatment. According to the Tukey test, bars with different lower-case letters indicate significant differences (*P* ≤ 0.05).

The quantity of extra-large-size fruits from FPM treatment was higher (*P* ≤ 0.05) among nematicide treatments and similar (*P* > 0.05) to the non-inoculated control (7.8 fruits). The lower (*P* ≤ 0.05) number of extra-large-size fruits was observed on FSF treatment (Fig. 2). The rest of the treatments resulted in a similar (*P* > 0.05) quantity of extra-large-size fruits when compared with the inoculated control (2.5 fruits) (Fig. 2). The two treatments with fluopyram (FPM and FZL+FPM) resulted in a higher (*P* > 0.05) quantity of large-size fruits. Both treatments were similar (*P* > 0.05) to the non-inoculated control (63.5 fruits). The FSF treatment produced a lower (*P* ≤ 0.05) number of large-size fruits (37.3 fruits) (Fig. 2). The numbers of medium-size fruits from FZL, FSF+FPM, and FZL+FPM treatments were higher (*P* > 0.05) when compared with the rest of the treatments. Moreover, the number of small fruits was higher with the FZL and the FSF treatments (*P* ≤ 0.05). The FPM treatment observed a lower quantity of small fruits (*P* ≤ 0.05) (Fig. 2).

**Figure 3.**
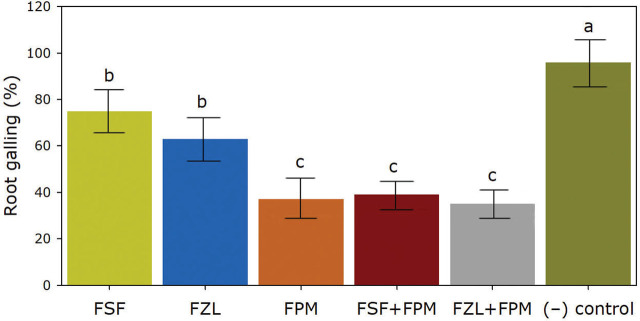
Root galling of tomato plants under fluorinated nematicide treatments for managing *Meloidogyne enterolobii*. FSF= fluensulfone, FZL= fluazaindolizine, FPM= fluopyram, FSF+FPM= fluensulfone plus fluopyram, FZL+FPM= fluazaindolizine plus fluopyram, (−) control= inoculated plants without nematicide treatment. No galling was found on the non-inoculated control treatment (+). Data are means ± standard errors of 168 plants per treatment. According to the Tukey test, bars with different lower-case letters are significantly different (*P* ≤ 0.05).

Tomato roots from the inoculated control without nematicide application showed 96% galling. All nematicide treatments reduced (*P* ≤ 0.05) the galling of tomato roots. This variable grouped (*P* > 0.05) the FSF and FZL treatments with a higher (*P* ≤ 0.05) root galling (63% and 75%, respectively) when compared with the rest of the treatments. Finally, FZL+FPM, FPM, and FSF+FPM showed lower (*P* ≤ 0.05) levels of root galling (35%, 37%, and 39%, respectively) (Fig. 3).

All nematicide treatments had a higher (*P* ≤ 0.05) plant height when compared with the inoculated control. The treatments FZL, and the combination of nematicides observed a plant height similar (*P* > 0.05) to the non-inoculated control (489 cm). However, tomato plants under nematicide treatments FSF and FPM showed a lower (*P* ≤ 0.05) height when compared with the non-inoculated control (Table 2).

**Table 2.** Growth variables of tomato plants under fluorinated nematicide treatments for managing *Meloidogyne enterolobii*.

Treatment*	**Plant height (cm)^yz^**	**Stem diameter (mm)**	**Plant fresh weight (kg)**	**Dry weight of roots (g)**
FSF	436 ± 18 b	13.41 ± 1.61 b	4.75 ± 0.12 b	67.00 ± 7.2 b
FZL	451 ± 13 ab	13.72 ± 1.47 b	5.29 ± 0.17 ab	61.44 ± 8.6 b
FPM	432 ± 16 b	14.68 ± 1.71 ab	4.75 ± 0.16 b	59.75 ± 10.5 c
FSF+FPM	469 ± 19 a	14.61 ± 1.63 ab	6.00 ± 0.27 ab	60.50 ± 7.9 bc
FZL+FPM	474 ± 13 a	15.33 ± 1.75 a	6.25 ± 0.31 ab	57.63 ± 11.8 c
(+) control	489 ± 24 a	16.08 ± 1.81 a	7.50 ± 0.28 a	60.75 ± 9.6 c
(−) control	340 ± 17 c	12.62 ± 1.72 b	2.25 ± 0.21 c	101.70 ± 12.6 a

x FSF= fluensulfone, FZL= fluazaindolizine, FPM= fluopyram, FSF+FPM= fluensulfone plus fluopyram, FZL+FPM= fluazaindolizine plus fluopyram, (+) control= non-inoculated plants without nematicide treatment, (−) control= inoculated plants without nematicide treatment.

y Data are means ± standard errors of 168 plants per treatment.

z Data in columns with different lower-case letters indicate significant differences according to the Tukey test (*P* ≤ 0.05).

The stem diameter from plants with FPM, FSF+FPM, and FZL+FPM were similar (*P* > 0.05) when compared with the non-inoculated control (16.08 mm). Only the FZL+FPM treatment showed a larger steam diameter (*P* ≤ 0.05) when compared with the inoculated control without nematicide application (15.33 and 12.62 mm, respectively) (Table 2).

All nematicide treatments had a higher (*P* ≤ 0.05) fresh plant weight when compared with the inoculated control without nematicide application (2.25 kg) (Table 2). Except for FPM and FSF, the fresh plant weight from nematicide treatments was similar (*P* > 0.05) to that of the non-inoculated control (7.5 kg).

The dry weight of roots in the non-inoculated control (60.75 g) was lower (*P* ≤ 0.05) when compared with the inoculated control without nematicide application (101.7 g). All nematicide treatments observed a lower dry weight of roots when compared with the inoculated control without nematicide application. Only the individual pre-plant nematicide treatments showed a higher dry weight of roots when compared with the non-inoculated control (Table 2).

## Discussion

In the present experiments, tomato crops were subjected to elevated population pressures of *M. enterolobii.* Therefore, the inoculated plants without nematicide application (negative control treatment) resulted in severe galling to the entire root system. Yield and fruit quality were reduced with a prevalence of medium-size and small-size fruits.

With the use of fluensulfone, root galling was reduced, and the loss in plant height and fresh weight variables were also reduced. However, a dramatic loss in numbers of extra-large-size and large-size fruits occurred. Tomatoes have previously been considered sensitive to phytotoxicity from fluensulfone at recommended doses (Giannakou and Panopoulou, 2019). Our application timing was at 12 DBP, and phytotoxicity symptoms were absent; however, fluensulfone has a slower nematicide activity and a more limited hatching inhibition than fluopyram (Oka et al., 2009; Oka and Saroya, 2019). The latter probably led our experiments to an out-of-phase of the duration of the nematicidal activity concerning the high population pressure by *M. enterolobii*.

The effect of fluazaindolizine on *Meloidogyne* spp. is slow without hatching inhibition or ovicidal activity; however, the residual and irreversible nematicidal effect on *Meloidogyne* J2 is very similar to fluensulfone (Thoden and Wiles, 2019; Wram and Zasada, 2019). In our experiments, fluazaindolizine visually suppressed root galling, loss of height, and fresh plant weight better than fluensulfone; however, both were statistically differentiated only by the number of large-size fruits. The latter showed that the duration of the nematicidal activity of fluazaindolizine was more synchronized than fluensulfone, and suggests the need for a higher amount of fluazaindolizine product or a higher number of applications (Qiao et al., 2020).

Among fluorinated nematicides, fluopyram affects nematodes more rapidly and has a higher effect, inhibiting egg hatching of *Meloidogyne* (Oka and Saroya, 2019). The latter could suggest higher effectiveness if several applications are used during the crop cycle; however, the dosage recommended by the manufacturer is not higher than the one applied here. At lower concentrations, the control is not obtained (Faske and Hurd, 2015; Oka and Saroya, 2019). In our study, fluopyram kept the dry root weight similar to the non-inoculated control and a low galling of roots. With fluopyram treatment, the number of extra-large-size fruits was not reduced; therefore, the timing of suppression of nematode invasion with fluopyram permitted an adequate fruit filling. The duration of the nematicidal activity of fluopyram, although correctly synchronized for yield and fruit quality protection, allowed a loss in plant height and weight and a reduction in the quantity of medium-size fruits.

The delay in fluopyram application timings in treatments with combinations of nematicides resulted in the loss of extra-large-size fruit production. However, nematicide combinations were more effective when compared with a single fluensulfone application. Moreover, with fluensulfone plus fluopyram and fluazaindolizine plus fluopyram treatments, the plant variables and yield were unaffected by *M. enterolobii*. Root damage was also controlled, allowing a regular production of large-size and medium-sized fruits. We conclude that fluopyram, fluensulfone, and fluazaindolizine nematicides have potential in the management programs of *M. enterolobii* on tomato crops. Fluopyram was more effective among individual nematicide treatments. The pre-plant applications of fluensulfone and fluazaindolizine reduced the damage levels on tomato roots by *M. enterolobii*; however, combining these with fluopyram allowed a better overall management of *M. enterolobii* tomato. These findings represent a valuable source for the design of strategies in *M. enterolobii* management programs in Sinaloa.
